# Intracisternal delivery of AAV9 results in oligodendrocyte and motor neuron transduction in the whole central nervous system of cats

**DOI:** 10.1038/gt.2014.16

**Published:** 2014-02-27

**Authors:** T Bucher, L Dubreil, M-A Colle, M Maquigneau, J Deniaud, M Ledevin, P Moullier, B Joussemet

**Affiliations:** 1 Laboratoire de Thérapie Génique, INSERM UMR U1089, Institut de Recherche Thérapeutique 1, Université de Nantes, Nantes Cedex 01, France; 2 INRA UMR U703, Animal Pathophysiology and Biotherapy for Muscle and CNS Diseases, Nantes, France; 3 Nantes-Atlantic College of Veterinary Medicine, Food Science and Engineering (Oniris), LUNAM University, Nantes Cedex 03, France; 4 Department of Molecular Genetics and Microbiology, College of Medicine, University of Florida, Gainesville, FL, USA

## Abstract

Systemic and intracerebrospinal fluid delivery of adeno-associated virus serotype 9 (AAV9) has been shown to achieve widespread gene delivery to the central nervous system (CNS). However, after systemic injection, the neurotropism of the vector has been reported to vary according to age at injection, with greater neuronal transduction in newborns and preferential glial cell tropism in adults. This difference has not yet been reported after cerebrospinal fluid (CSF) delivery. The present study analyzed both neuronal and glial cell transduction in the CNS of cats according to age of AAV9 CSF injection. In both newborns and young cats, administration of AAV9-GFP in the cisterna magna resulted in high levels of motor neurons (MNs) transduction from the cervical (84±5%) to the lumbar (99±1%) spinal cord, demonstrating that the remarkable tropism of AAV9 for MNs is not affected by age at CSF delivery. Surprisingly, numerous oligodendrocytes were also transduced in the brain and in the spinal cord white matter of young cats, but not of neonates, indicating that (i) age of CSF delivery influences the tropism of AAV9 for glial cells and (ii) AAV9 intracisternal delivery could be relevant for both the treatment of MN and demyelinating disorders.

## Introduction

Adeno-associated viral (AAV) gene therapy holds great promise for the treatment of neurodegenerative disorders.^1^ However, local administration usually restricts gene expression in the targeted cerebral structures and does not allow widespread gene delivery to the central nervous system (CNS), especially in glial cells,^2, 3, 4^ yet implicated in several neurological diseases or CNS injuries.^5, 6, 7, 8^

Systemic AAV serotype 9 (AAV9) delivery has been shown to efficiently transduce the whole spinal cord in neonatal mice and kittens^9,10^ and to increase the life expectancy of neonatal mouse models of spinal muscular atrophy.^11, 12, 13^ In adult mice, the results differ according to the studies,^14^ but preferential glial cell transduction has been reported in adults in contrast to neonates injected intravenously (IV) with AAV9,^9^ suggesting that age at the times of injection could potentially influence the neurotropism of the vector and the efficiency of motor neuron (MN) and glial cell transduction. In adult primates, AAV9 targeted also preferentially astrocytes and less efficiently neurons when it was administered IV.^14,15^

Recently, delivery of AAV9 in the cerebrospinal fluid (CSF) was shown to target neurons throughout the CNS, including the brain, spinal cord or dorsal root ganglia, in mice and in large animals, that is, primates, dogs and pigs.^16, 17, 18, 19, 20, 21^ However, the influence of age at the times of CSF delivery on MN and glial cell transduction is not yet clearly determined.

The purpose of this study was to determine the proportion of transduced MNs all along the spinal cord and the profile of glial cell transduction in the whole CNS after intracisternal (IC) injection of self-complementary (sc) AAV9-CMV (cytomegalovirus)-GFP (green fluorescent protein) vectors in both neonatal and young cats, a large animal model in which different neurodegenerative diseases^22, 23, 24, 25, 26^ and MN degeneration^27^ have been described. Our results showed that scAAV9 injected in the CSF transduced the vast majority of MNs all along the spinal cord (84±5% in the cervical, 99±1% in the lumbar), whatever the age at the times of injection, with a limited off-target biodistribution of the vector. Surprisingly, in addition to a significant transduction of neurons in the spinal cord and in various brain structures, an important proportion of oligodendrocytes was also found to express GFP in the spinal cord and brain white matter, but only in young cats. These results indicate a particular tropism of AAV9 for oligodendrocytes when it is administered by IC delivery after the neonatal period that in turn may have important consequences, especially for the treatment of demyelinating diseases affecting the whole CNS.

## Results and discussion

**Figure 1:**
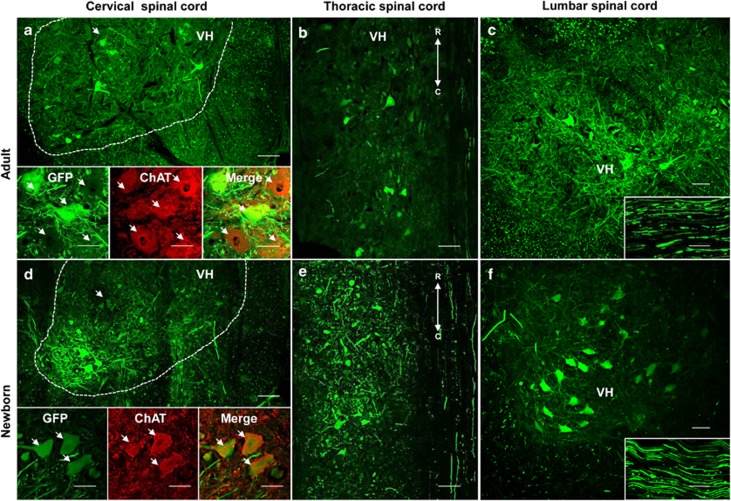
AAV9 displays an important tropism for motor neurons after CSF delivery in cats that is not affected by age of injection. Transverse sections of the cervical ventral horn (VH) from the spinal cord from respectively young cat C5 (**a**) or newborn cat C1 (**d**) observed by laser scanning confocal microscopy 30 days after the injection of 10^12^ vg kg^−1^ of scAAV9-CMV-GFP into the cisterna magna (arrows: GFP positive MNs). Scale bar=100 μm. High magnification of cervical motor neurons (MNs) from the young cat C5 (**a**, inset) or the newborn cat C1 (**d**, inset) with ChAT immunolabeling. Colocalization of ChAT immunolabeled MNs with GFP native fluorescence in MNs was observed in ventral horn (arrows). Scale bar=50 μm. Horizontal section of the VH from the thoracic spinal cord from the young cat C5 (**b**) or the newborn cat C1 (**e**) observed by laser scanning confocal microscopy showed homogenous expression of GFP in MNs all along the spinal cord. Scale bar=100 μm. R: rostral orientation; C: caudal orientation. Transverse sections of the VH from the lumbar spinal cord from the young cat C5 (**c**) or the newborn cat C1 (**f**) observed by laser scanning confocal microscopy. Scale bar=100 μm. Longitudinal sections of the sciatic nerve from the young cat C5 (**c**, inset) or the newborn cat C1 (**f**, inset) observed by laser scanning confocal microscopy. Scale bar=100 μm.

To determine glial cell and MN transduction profiles in the CNS vs age of AAV9 CSF delivery in cats, three 2-day-old kittens (C1, C2, C3) and three 7-week-old young cats (C4, C5, C6) were injected with 10^12^ viral genomes (vg) kg^−1^ of scAAV9-CMV-GFP in the cisterna magna and killed at 1 month post injection. A GFP signal was observed in the dorsal and ventral parts of the spinal cord in both neonates and young cats (Figure 1). In the dorsal part, the GFP signal was found in the dorsal columns, especially in the axons of the gracile and cuneate fasciculi identified by neurofilament immunostaining (data not shown), suggesting that sensory neurons of the dorsal root ganglias were efficiently targeted by the vector after CSF delivery.

In the ventral horn, the choline acetyltransferase (ChAT) immunostaining demonstrated that a large number of MNs expressed the GFP (Figure 1a and insets in young cats; Figure 1d and insets in neonates and Supplementary Figure S1) homogeneously all along the ventral horn from the cervical to the lumbar part (Figures 1a–f and Supplementary Figure S1). Moreover, the GFP signal was also detected in the axons of the sciatic nerve in both neonates and young cats, indicating a transport of the GFP protein all along the peripheral axonal pathway (Figure 1c, inset in young cats; Figure 1f, inset in neonates).

The GFP-positive MNs were scored in cervical and lumbar enlargements that innervate upper and lower limbs, respectively. MN quantification revealed that 86.1±6.8 and 82.7±7.4% of the lower MNs expressed GFP in the cervical enlargement of the injected neonates and young cats, respectively (Table 1, expressed as mean ±s.e.m., no statistical difference: *P*>0.05). The number of transduced MNs was slightly increased in the lumbar enlargement with 99.9±0.1% (neonates) and 97.9±1.2% (young cats) GFP-positive MNs. A lower number of GFP-positive MNs was found in the cervical spinal cord of two cats (51.4% for the neonate cat C1 and 48.2% for the young cat C5) compared with the other cats (100% and 98.7% for the neonate cats C2 and C3, respectively, 100% for both the young cats C4 and C6). This disparity was not observed in the lumbar spinal cord where >95% of MNs expressed GFP in all the cats. These data suggest that AAV9 vector targeted more efficiently MNs of the lumbar than of the cervical regions after IC delivery in some animals. These data could be explained by the flow of CSF secreted in the cerebral ventricles and driven to the cauda equina, allowing AAV particles, injected in cisterna magna, to be transported to the lumbar spinal cord with an efficiency and a diffusion rate that probably vary according to the conditions of AAV injection, the volume, the flow rate or the position of the animal.

On the other hand, no statistical difference (*P*>0.05) was found between IC injections in neonates vs in young cats in contrast to data previously obtained after IV injections in mice,^9,11^ indicating that the dispersion and the tropism of AAV9 for MNs was not affected by the animal age after IC injection. This difference could be related to the route of administration as the IC injection concentrates the AAV particles in the nervous tissue. Besides, the proportion of transduced MNs was higher in this study than those previously detected after IV injection in newborn cats (transduced MNs: ≈40%) and in young cats (transduced MNs: ≈15%).^10^

Moreover, GFP-specific real-time quantitative PCR performed in the spinal cord of the newborns (C1, C2, C3), as well as in the spinal cord and the peripheral organs of the young cats injected in the cisterna magna (C4, C5 and C6) and in three other young cats (C7, C8, C9) injected with 10^12^ vg kg^−1^ of scAAV9-CMV-GFP in the jugular vein showed higher number of vg in the spinal cord after IC delivery (0.07±0.01 in newborns, 0.38±0.04 vg dg^−1^ in young cats for IC vs 0.02±3 × 10^−3^ vg dg in young cats for IV; 10^−3^±7 × 10^−4^ vg dg^−1^ for uninjected cat; comparison between IC and IV-injected young cats: *P*<0.005) and a lower dispersion of the vector to the periphery compared with the systemic administration of the vector in young cats (2.3±0.8 vg dg^−1^ in the liver for IC vs 28.1±16.2 vg dg^−1^ for IV, 0.01 vg dg^−1^ for uninjected cat; Supplementary Figure S2). These results suggest that a large number of viral particles penetrated and persisted in the spinal cord after IC delivery compared with IV injection with a limited off-target biodistribution of the vector in systemic organs, for instance, the liver, consistent with recent studies performed in other large animal models.^18,20,28^

**Figure 2:**
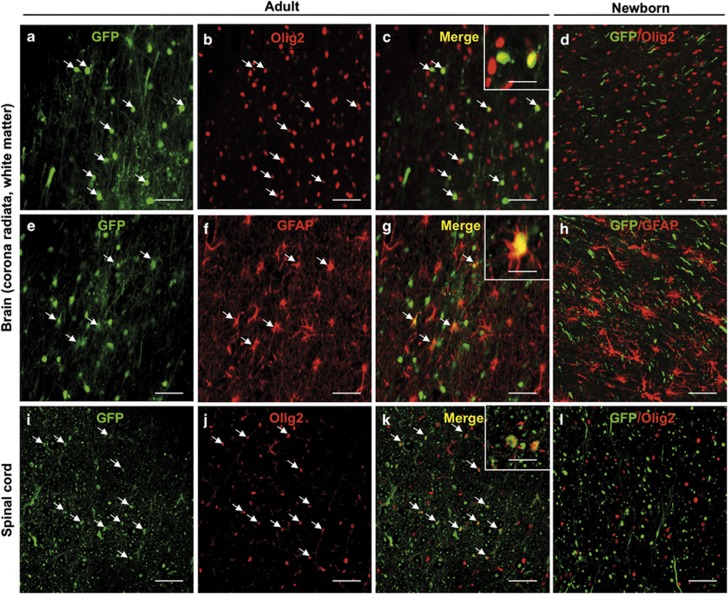
Intracisternal delivery of scAAV9-CMV-GFP mediates transgene expression in oligodendrocytes throughout the brain and the spinal cord white matter in injected young cats. Coronal sections of the white matter in the brain, that is, corona radiata from temporal and occipital lobes, (**a**–**h**) or in the spinal cord (**i**–**l**) with GFP native fluorescence (**a**, **e**, **i**), Olig2 staining (**b**, **j**), GFAP staining (**f**) or merged images of Olig2 staining with direct GFP fluorescence (**c**, **d**, **k**, **l**) and merged images of GFAP staining with direct GFP fluorescence (**g**, **h**), 30 days after the injection of 10^12^ vg kg^−1^ of scAAV9-CMV-GFP into the cisterna magna of the young cat C5 (**a**–**c**, **e**–**g**, **i**–**k**) or the newborn cat C1 (**d**, **h**, **l**) (arrows: GFP-positive oligodendrocytes and GFP-positive astrocytes). Scale bar=20 μm.

To determine the nature of the transduced cells throughout the CNS, the brains of the injected cats were also examined for GFP expression. The vector transduced neurons in the cerebellum, thalamus, striatum, motor and sensory cortex of both newborn (data not shown) and young injected cats, as we previously showed.^29^ In addition to GFP-positive neurons, transduced astrocytes were also seen in the cerebral cortex of newborn and young cats (Figure 2), indicating that IC injection of AAV9 resulted in both neuronal and astrocytic transduction in the gray matter of the brain, consistent with recent reports in primates^17,18^ or in dogs.^21^ More surprisingly, glial fibrillary acidic protein (GFAP)-positive fibrous astrocytes in the brain and a significant number of oligodendrocytes throughout both the corona radiata in the brain and the white matter all along the spinal cord expressed GFP (Figure 2). Transduced oligodendrocytes were found in different brain regions from the frontal to the occipital lobes and in the brainstem (Figure 2 and Supplementary Figure S3). This important transduction of oligodendrocytes in both the brain and the spinal cord has been found only in the young cats injected after the neonatal period. In contrast, cats injected during the neonatal period did not exhibit GFP-positive oligodendrocytes in the spinal cord and brain white matter.

This difference of AAV9 tropism for glial cells between neonates and young cats could be related to AAV9 receptors. Indeed, AAV9 uses β-galactose as a receptor to transduce many different cell types *in vitro*,^30^ and tissue glycosylation profiles was shown to affect AAV9 tropism.^31^ Tissue glycosylation level could therefore explain the different transduction profiles we observed in the CNS of newborn and young cats after AAV9 IC delivery. To investigate this hypothesis, we cut the brain of a 10-day-old newborn kitten C10 and the brain of a 3 year-old adult cat C11 and used rhodamine-conjugated Ricinus Communis Agglutinin I, known to bind specifically to terminal β-galactose residues and therefore described to inhibit binding of AAV9.^32,33^ We observed a significant reduction of β-galactose staining, especially in the white matter of brain, in the 10-day-old kitten compared with the adult cat brain (Supplementary Figure S4; 7.5±1.1 Mean Gray Value for the neonate C10 vs 15.1±0.9 Mean Gray Value for the adult cat C11; *P*<0.001 by Mann–Whitney *U*-test). These data suggest that the absence of transduced oligodendrocytes in the brain white matter of neonatal kittens could be explained in part by a smaller number of accessible AAV9 receptors in brain tissue of neonatal kittens compared with brain tissue of older cats.

Another explanation could be related to the CSF volume, higher with regard to the body weight in neonate compared with juveniles that could therefore result in different vector concentration within the CNS.

The tropism of AAV9 for oligodendrocytes has never been described by other studies using CSF delivery of the vector in mice and different large animals.^16, 17, 18, 19, 20, 21^ The purity of AAV vector preparations could potentially explain this particular tropism. Indeed, cesium chloride (CsCl)-purified AAV vectors have been described to contain large amount of impurities^34^ and to display an astroglial transduction pattern in contrast to the expected neuronal expression.^35^ The authors hypothesized that protein impurities obtained after the CsCl purification could induce astrogliosis that could stimulate transgene expression in astroglial cells and inhibit neuronal transduction. Otherwise, dilution of the vector particles, variation in particles conformation or active interference with vector uptake could also affect the neuronal tropism of the vector in relation to the degree of protein impurities or the method of purification.

In this study, we used an optimized CsCl protocol^34^ resulting in higher AAV vector purity that could induce high transduction efficiency both in neurons and oligodendrocytes after CSF delivery of the vector.

The promoter used, that is, the CMV promoter, may also impact on the number and the nature of the transduced cells within the CNS after CSF injection. Indeed, in studies describing neuronal transduction after IV or CSF delivery of AAV9, different types of promoter have been used, including CBA (chicken beta actin), CMV or CBh (chicken beta actin short), and there is increasing evidence that the choice of the promoter is critical for transgene expression in neuronal or glial cell types. The CMV promoter has been described as poorly effective in inducing transgene expression in astrocytes^16^ in concordance with our results, especially in neonatal kittens. On the other hand, the CMV promoter has been shown to mediate higher transgene expression in MNs than the CBA promoter.^16,19^ Nevertheless, in large animals, only CBA and CBh promoters were used when AAV9 was delivered in the CSF.^17,18,20,21^ The combination of a CMV promoter and a new method of CsCl purification may thus explain in cats the specific tropism of the vector described in this study, that is, the transduction of both oligodendrocytes and MNs with a high efficiency.

Futhermore, our study indicates that IC delivery of AAV9 could be used to mediate high transduction of oligodendrocytes in the whole CNS in contrast to AAV intracerebral injections that usually target a small number of oligodendrocytes locally.^36,37^

Finally, to investigate the immunogenicity induced by AAV9 IC delivery, we quantified the AAV9 capsid-neutralizing antibodies in the serum and in the CSF of injected cats at the time of killing. The IC route systematically resulted in AAV9 antibodies in the sera with a titer varying between 1/10 and 1/2000, independently of the age of the animal (Table 2). In contrast, AAV9 antibodies were detected in the CSF of only one young cat (cat C5), at low level (1/20, Table 2). Interestingly, the cat C5 did not exhibit the highest titer of AAV9-neutralizing antibodies in the serum but showed a large number of vector copies in the periphery, especially in the liver (five vector copies per diploid genome, Supplementary Figure S2). These results may suggest that (i) the level of AAV9-neutralizing antibodies in the serum does not predict for the presence of anti-AAV9 antibodies in the CSF and (ii) a significant number of vg in the periphery after CSF delivery of the vector might elicit a production of AAV9-specific antibodies within the CNS.

These results also confirm recent data obtained in dogs that also display asymmetrical distribution of AAV-neutralizing antibodies across the blood–brain barrier, with high serum-neutralizing antibody titers and low CSF-neutralizing antibody titers after IC delivery of AAV9 vector.^21^ Taken together, these results suggest that it could be possible to re-inject AAV9 vectors in the CSF of the majority of animals that received an initial IC injection of the vector. This hypothesis is also consistent with recent data showing spinal cord transduction after intrathecal delivery of AAV9 vector in non-human primates or in dogs despite the presence of pre-existing immunity against AAV.^21,38^

We also investigated the presence of GFP-specific antibodies in the serum of injected animals at 4 weeks post-injection by western blotting. The presence of small amounts of GFP-specific antibodies in the serum of a young cat (C6) and of the three injected newborn kittens (C1, C2 and C3; Supplementary Figure S2) indicated that IC injection did not prevent the development of immune responses against the immunogenic GFP protein. Further investigation may therefore be necessary to ensure long-term expression of potential therapeutic transgenes in the CNS of large animal models after CSF delivery of AAV9 vector.

Altogether, the results of this study highlight the potential of IC AAV9 delivery to transfer therapeutic gene to MNs and oligodendrocytes in the whole CNS providing a potential application for the treatment of both MN and demyelinating diseases.

## Materials and methods

### Vectors

Pseudotyped AAV9 vectors were generated by packaging AAV2-based recombinant sc genomes in AAV9 capsids^39^ purified by double CsCl gradient as previously described,^34^ at the University Hospital of Nantes (http://www.vectors.nantes.inserm.fr). The AAV2 plasmids contained the gene encoding GFP under the control of CMV promoter in a sc genome. Physical particles were quantified by dot blot hybridization, and vector titers were expressed as vector genomes per milliliter.

### *In vivo* AAV injections

Breeding and injected healthy cats were housed in the Boisbonne Center at Nantes Veterinary School (ONIRIS, Nantes, France). Experiments were approved by the Regional Ethics Committee and were carried out according to the European guidelines for the care and use of experimental animals.

Three 2-day-old healthy kittens (C1, C2, C3) and three 7-week-old healthy young cats (C4, C5, C6) were injected with 10^12^ vg kg^−1^ of scAAV9-CMV-GFP in the cisterna magna with a volume of 1 ml kg^−1^ under general anesthesia obtained by isoflurane inhalation (3% v/v) and morphine injection (0.2 mg kg^−1^). Neonatal kittens weighed between 100 and 200 g and received between 0.1 and 0.2 ml of AAV9 vector (concentration: 10^12^ vg ml^−1^ in Dulbecco's phosphate-buffered saline (PBS)). Seven-week-old cats weighed between 1 and 1.5 kg and received 1–1.5 ml of the vector at the same concentration. In all, 0.1–0.2 ml of CSF in newborns and approximately 1 ml of CSF in young cats were removed before the injection of the vector to avoid any risk of intracranial hypertension. No side effect possibly related to surgery or vector deposits was noticed. The absence of AAV9-neutralizing antibodies was checked in the serum of 7-week-old cats and in the serum of mothers of neonatal kittens, before injection. For IV injections, 10^12^ vg kg^−1^ of scAAV9-CMV-GFP in a volume of 1 ml kg^−1^ were injected into the jugular vein of three 7-week-old healthy young cats (C7, C8 and C9).

### Tissue preparation

One month following IC or IV injection, animals were anesthetized (150 μg kg^−1^ medetomidine, 10 mg kg^−1^ ketamine, 0.2 mg kg^−1^ morphine) and perfused transcardially with 10 ml PBS followed by 100 ml 4% paraformaldehyde. Brains and spinal cords were removed and cut into coronal 5-mm blocks, post-fixed by incubation in 4% paraformaldehyde and cryoprotected by overnight incubation in 30% sucrose. Samples were embedded in optimal cutting temperature compound, frozen on dry ice and cryostat sectioned (10 μm).

### GFP expression, immunostaining and MN quantification

Spinal cord and brain slices were examined for GFP expression using a laser scanning confocal microscope (Nikon, C1, Champigny sur Marne, France) equipped by a blue argon ion laser at 488 m. For ChAT immunofluorescence, the brain and spinal cord sections were blocked by incubation with 1% rabbit serum (Dako, Les Ulis, France) in 0.4% Triton X-100 in PBS and incubated overnight at room temperature with a goat polyclonal anti-ChAT antibody (1:100, Chemicon International, Temecula, CA, USA); the sections were then incubated with a biotinylated rabbit anti-goat antibody (1/300, Dako). For GFAP and Olig2 immunostaining, the brain and spinal cord sections were incubated overnight at room temperature with antibodies directed against GFAP (1/4000, Dako) or Olig-2 (1/100, Millipore, Billerica, MA, USA) followed by a biotinylated rabbit anti-goat antibody (1/300, Dako). Sections were then incubated with Alexa fluor 555-conjugated streptavidin (Life Technologies, Saint Aubin, France), mounted in Mowiol (Calbiochem, San Diego, CA, USA) and scanned serially using the argon ion laser (488 nm) to observe GFP signals and with a helium neon laser (543 nm) to observe Alexa fluor 555 signals. Each image was recorded in a separated channel (channel green for GFP and channel red for Alexa fluor 555) and overlayed to allow detection of colocalized fluorescent signals.

For MN quantification, the number of Nissl-stained (Neurotrace Molecular Probes, Life Technologies) and GFP-positive MNs was estimated relative to the total number of MNs using the NIS-Elements AR Image analyzer (Nikon) on 10-μm thick sections (two sections containing two ventral horns for each cervical and lumbar enlargement). The count of GFP-positive MNs was performed for each cat using four ventral horns of both cervical and lumbar intumescences. For each horn, an average of 15–20 MNs were counted, resulting in a total of 60–80 MNs for each region and approximately a total of 140 MNs per cat.

Furthermore, a 7.2 mm length horizontal section was performed in the thoracic spinal cord of the newborn cat C1 and of the young cat C5 to verify the homogeneous distribution of GFP-positive MNs throughout the spinal cord.

### Galactose residue staining in feline brain tissue

The brains of two cats—a neonatal kitten C10, who died 10 days after birth, and an adult cat C11 killed at the age of 3 years—were used to stain β-galactose residues in feline CNS tissue. Brains were cut into coronal 5-mm blocks, frozen on dry ice and cryostat-sectioned (10 μm). Tissue cryosections were incubated for 1 h at 4 °C with blocking buffer and rhodamine ricinus communis agglutinin RCA_120_ (Vector Laboratories, Burlingame, CA, USA) was added at 100 μg ml^-1^ in blocking buffer or blocking solution alone for control and incubated at 4 °C for 15 min^32^. The lectine sol ution was then removed, and sections were washed in PBS and mounted in Mowiol medium.

### Quantitative PCR Analysis

Genomic DNA was extracted from frozen tissues of the newborns (C1, C2, C3) and of the young cats (C4, C5 and C6) injected in the cisterna magna and of three other young cats (C7, C8, C9) injected with 10^12^ vg kg^−1^ of scAAV9-CMV-GFP in the jugular vein as previously described.^29^ Real-time PCR was conducted in duplicate with a LightCycler 2.0 instrument (Roche Diagnostics, Rotkreuz, Switzerland) using 50 ng of DNA in a 20-μl volume of the following solution: 10 μl Premix ExTaq (Takara Bio Inc., Shiga, Japan), 0.4 μl 10 μM Primer, 0.4 μl 10 μM FAM/TAMRA TaqMan probe, 5 μl template, and 3.8 μl H2O. The feline β-glucuronidase (βGLU) gene was used as a reference, and for each sample, Ct values were compared with those obtained with plasmid standard dilutions containing GFP and βGLU cDNA sequences. The ratio between transgene (GFP sequence) and genomic DNA (βGLU sequence) copy number provided the amount of transgene copy per cell. Primers and probe for GFP were : forward primer 5′-ACTACAACAGCCACAACGTCTATATCA-3′, reverse primer 5′-GGCGGATCTTGAAGTTCACC-3′, probe 6FAM5′-CCGACAAGCAGAAGAACGGCATCA-3′-TAMRA, and for feline βGLU : forward primer 5′-ACGCTGATTGCTCACACCAA-3′, reverse primer 5′-CCCCAGGTCTGCTTCATAGTT-3′, probe 6FAM5′-CCGGCCCGTGACCTTTGTGA-3′-TAMRA. Samples were heated at 95 °C for 20 s followed by 45 cycles of 95 °C for 10 s, 60 °C for 40 s. Control tissues extracted separately from vector-treated tissues were checked for GFP amplification. The sensitivity of the quantitative PCR is 10^−3^ copies/cell or 100 copies/50 ng of genomic DNA.

### Detection of AAV9-specific antibodies and GFP-specific antibodies

AAV9-specific antibodies in the serum and CSF of injected and uninjected cats were detected according to a previously described procedure.^40^ Briefly, serum or CSF samples were heat-inactivated at 56 °C for 35 min. Recombinant ssAAV2/9.CMV.LacZ (4 × 10^3^ vg/well) was diluted in serum-free Dulbecco's modification of Eagle's medium (DMEM, Sigma Aldrich, Saint Quentin Fallavier, France) supplemented with 2% fetal bovine serum (Eurobio, Les Ulis, France) and incubated with serial dilutions (initial dilution, 1:10) of heat-inactivated serum or CSF samples on DMEM for 20 min at 37 °C. Subsequently, sample vector mixture was added to 96-well plates containing 2 × 10^5^ Hela T cells/well that had been infected 2 h earlier with wild-type HAdV5 (8 viral particles/cell). After an incubation of 24 h at 37 °C and 5% CO2, cells were fixed with 0.5% glutaraldehyde and developed with X-Gal solution (Promega, Madison, WI, USA). The antibody titer was reported as the highest serum or CSF dilution that inhibited ssAAV2/9.CMV.LacZ transduction (β-galactosidase expression) by 100%.

GFP-specific antibodies were detected by western blotting. Briefly, following electrophoresis of 200 ng of recombinant GFP (Millipore, Billerica, MA, USA) per lane, proteins were transferred to a nitrocellulose membrane (Hybond, Amersham, Les Ulis, France) blocked with a solution of PBS supplemented with 5% non-fat dry milk, 1% NP-40 and 0.1% Tween 20 overnight at 4 °C. Membranes were then immunoblotted for 2 h with the sera of injected animals diluted at 1:100 and next incubated with peroxidase-labeled goat anti-cat immunoglobulin G (1:15000, Serotec, Oxford, UK) for 45 min. Rabbit anti-GFP antibodies (Millipore, Temecula, CA, USA) served as a positive control. All membranes were visualized using ECL Hyperfilm (GE Healthcare, Velizy-Villacoublay, France) and exposure to ECL Hyperfilm.

### Statistical analyses

Data were expressed as means±s.e.m. Statistical comparisons between the experimental groups were performed by the Mann–Whitney *U*-test. *P*-values <0.05 were considered statistically significant.

## Figures and Tables

**Table 1:** Proportion of spinal transduced MNs in cats injected with scAAV9-CMV-GFP by intracisternal route

*Age at injection*	*Cat*	*Proportion of GFP-positive MNs*	
		*Cervical enlargement*	*Lumbar enlargement*
2 days (newborn)	C1	51.4±3.3	100
	C2	100	100
	C3	98.7±1.3	99.7±0.3
50 days (young)	C4	100	100
	C5	48.2±3.2	95.6±2.3
	C6	100	100

Abbreviations: C, cat; CMV, cytomegalovirus; GFP, green fluorescent protein; MN, motor neuron; scAAV, self-complementary adeno-associated virus.

Results are expressed as means± s.e.m.

**Table 2:** Anti-AAV9 antibodies in cats injected with scAAV9-CMV-GFP by intracisternal route

*Age at injection*	*Cat*	*Anti-AAV9 antibodies*		
		*In serum*		*In CSF*
		*Before inj.*	*After inj.*	*After inj.*
2 days (newborn)	C1	NA	1/100	<1/10
	C2	NA	1/1000	<1/10
	C3	NA	1/2000	<1/10
50 days (young)	C4	<1/10	1/10	<1/10
	C5	<1/10	1/100	1/20
	C6	<1/10	1/500	<1/10

Abbreviations: AAV, adeno-associated virus; C, cat; CMV, cytomegalovirus; CSF, cerebrospinal fluid; GFP, green fluorescent protein; inj., injection; NA, not available; sc, self-complementary.
