# Supplementary figures and images for: Intracisternal delivery of AAV9 results in oligodendrocyte and motor neuron transduction in the whole central nervous system of cats
Source: Gene Ther. 2014 Feb 27;21(5):522–8. doi: 10.1038/gt.2014.16 (PMC4015314; doi:10.1038/gt.2014.16)

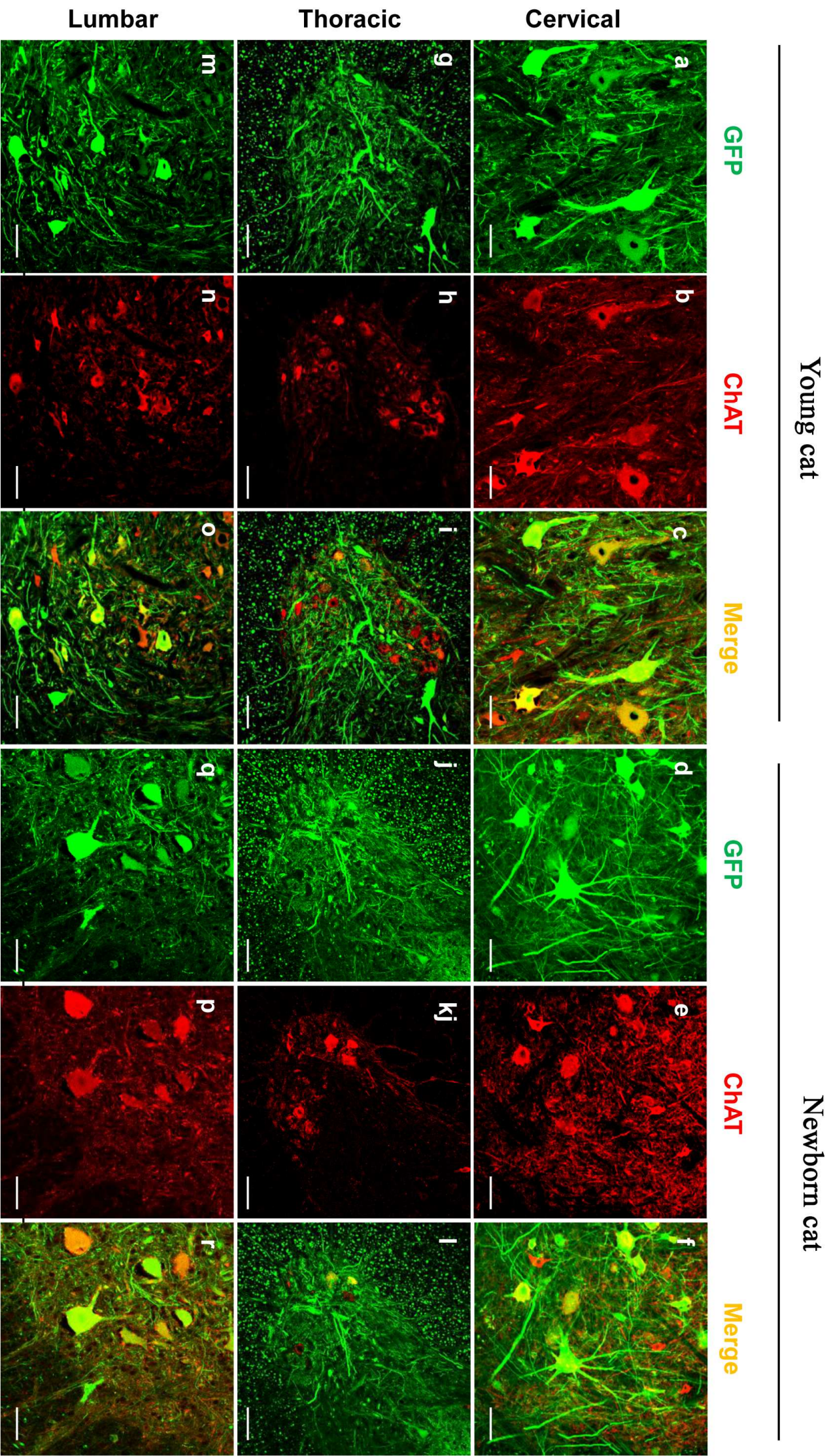

Supplement: Supplementary Figure S1 [file gt201416x1.pdf]

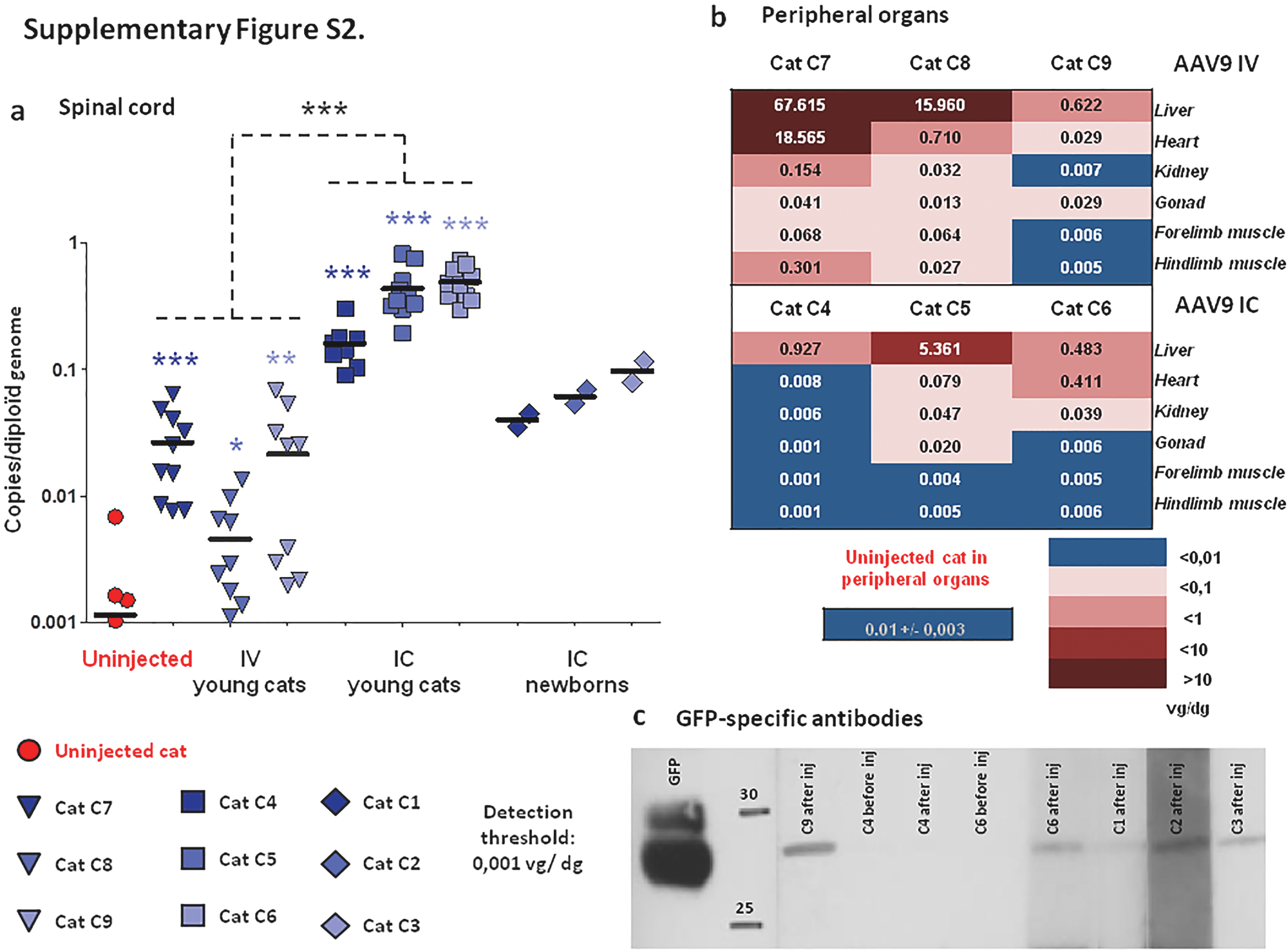

Supplement: Supplementary Figure S2 [file gt201416x2.tif]

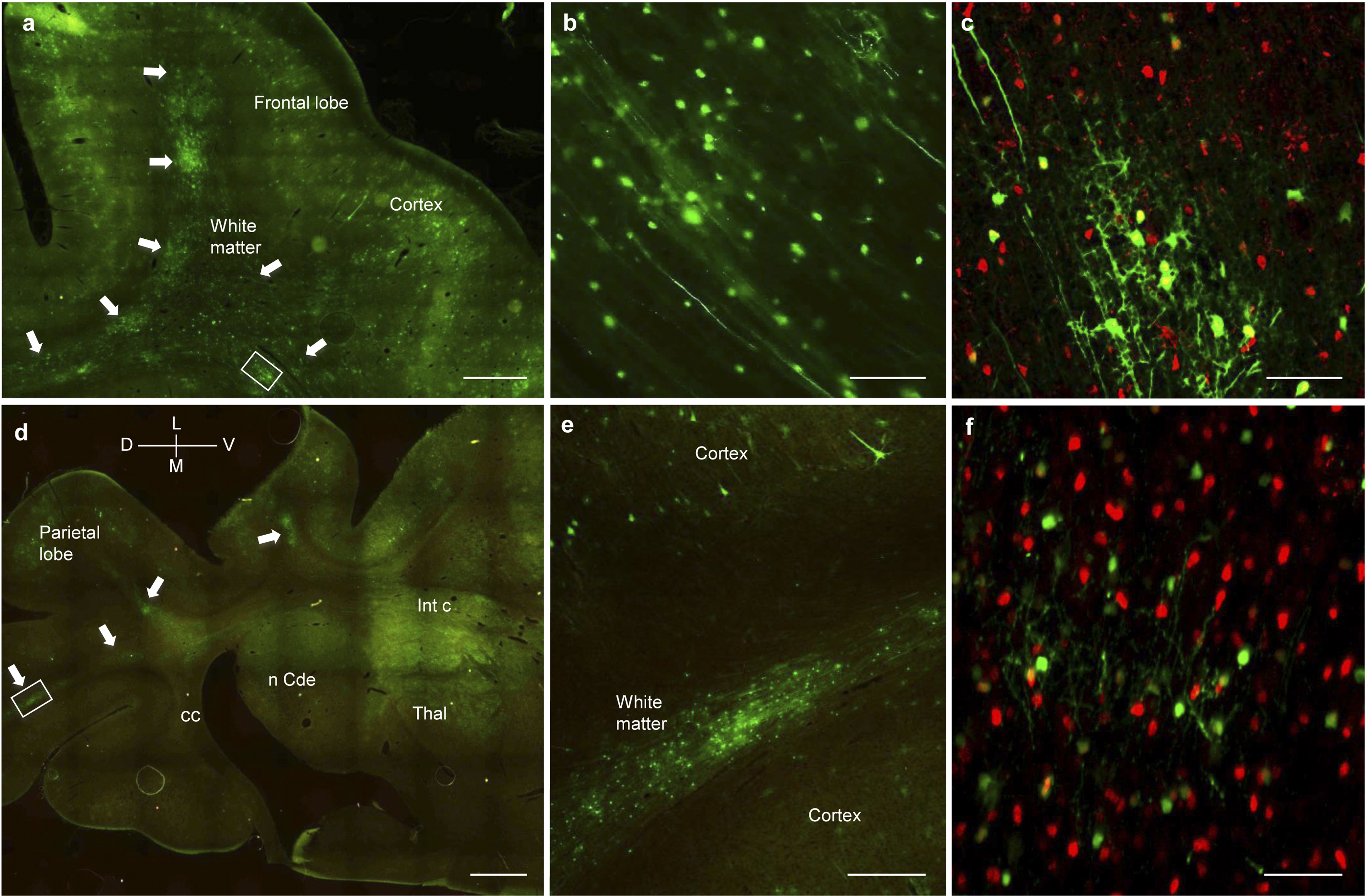

Supplement: Supplementary Figure S3 [file gt201416x3.tif]

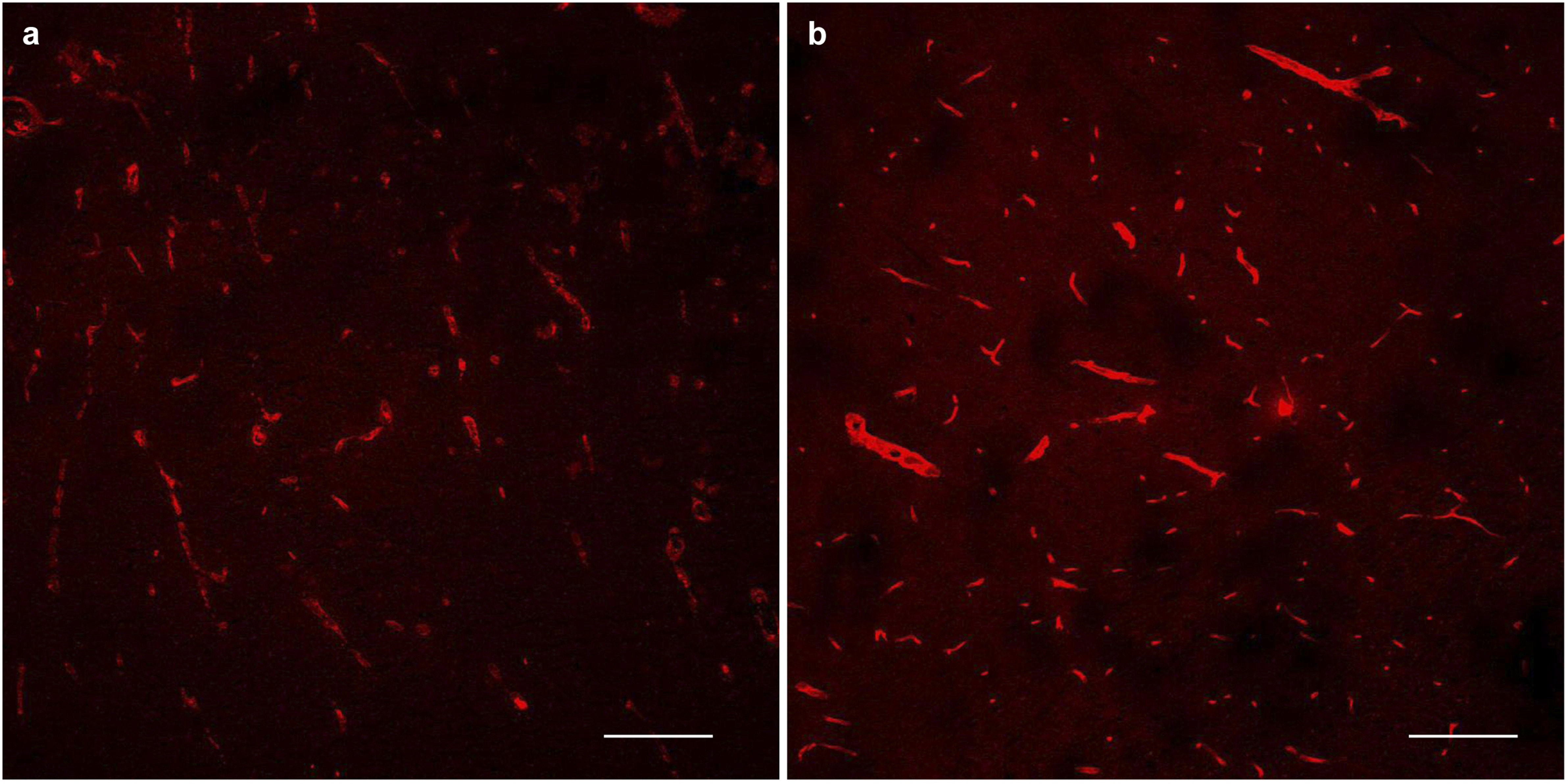

Supplement: Supplementary Figure S4 [file gt201416x4.tif]
