# Supplementary material for: Intracisternal delivery of AAV9 results in oligodendrocyte and motor neuron transduction in the whole central nervous system of cats
Source: Gene Ther. 2014 Feb 27;21(5):522–8. doi: 10.1038/gt.2014.16 (PMC4015314; doi:10.1038/gt.2014.16)
Supplement: Supplementary Figure Legends [file gt201416x5.doc]

**Supplementary Figure S1. Colocalisation of transduced MN and ChAT immunostaining in spinal cord.** Transverse sections of the ventral horn from the spinal cord of a young cat C4 and a newborn cat C2 with ChAT immunolabeling observed by laser scanning confocal microscopy. Colocalisation of ChAT immunolabeled MNs with GFP native fluorescence was observed in ventral horn all along the spinal cord, cervical (a-f), thoracic (g-l), lumbar (m-r) for both young and newborn cats, scale bar 100µm.

**Supplementary Figure S2. scAAV9-CMV-GFP** **intracisternal delivery mediates higher number of viral genomes in the central nervous system and lower dispersion in periphery compared to systemic scAAV9-CMV-GFP delivery but induces GFP-specific antibodies.**

Results of GFP-specific real-time quantitative PCR in the spinal cord (**a**) or in peripheral organs (**b**) of 3 young cats (cats C7, C8 and C9) injected in the jugular vein with 10^12^ vg/kg of scAAV9-CMV-GFP, 3 young cats (cats C4, C5 and C6) and 3 newborn kittens (C1, C2, C3) injected in the *cisterna magna* with 10^12^ vg/kg of scAAV9-CMV-GFP. Data were expressed in number of viral genomes (vg) per diploid genome. Differences between IV and IC delivery in young cat groups were analyzed using Mann Whitney test. *p* values <0.05 were considered significant.* p<0.05 ; ** p<0.01, *** p<0.005. vg, viral genome ; dg, diploid genome ; IV, intravenous ; IC, intracisternal. (**c**) Detection of GFP-specific antibodies by Western blot. Following electrophoresis of 200ng of GFP protein per lane, proteins were transferred to a nitrocellulose membrane and immunoblotted with the serum of the injected cats, taken before (young cats) and 4 weeks after vector injection (inj). The serum of C9 cat after the IV injection of AAV9-CMV-GFP vector served as positive control.

**Supplementary Figure S3. Profile of oligodendrocyte transduction in cat brain.**

(a) Coronal section of the frontal lobe, scale bar 1mm ; transduced oligodendrocytes are delineated by arrows, the rectangle corresponds to (b), scale bar 100 µm. (b) and (c) frontal sub-cortical white matter with GFP native fluorescence (b) and olig2 staining (c), scale bar 50 µm.

(d) Coronal section of the brain at the level of caudate nucleus (n Cde) and thalamus (Thal), scale bar 2mm; clusters of transduced oligodendrocytes are delineated by arrows, the rectangle corresponds to (e). D: dorsal orientation; V: ventral orientation; M: medial orientation, L: lateral orientation; cc: corpus callosum, Int c: internal capsule. (e) and (f) parietal sub-cortical white matter with GFP native fluorescence (e) scale bar 200 µm and olig2 staining (f), scale bar 50 µm.

**Supplementary Figure S4. Galactose staining in neonal and adult cats.** Difference in ß-galactose receptor expression between neonate (a) and adult (b) cats after RCA staining of the brain parietal white matter. Scale bar 200µm.
